# Substance use disorders and medical comorbidities among high-need, high-risk patients with diabetes

**DOI:** 10.1016/j.drugalcdep.2018.01.008

**Published:** 2018-03-03

**Authors:** Li-Tzy Wu, Udi E. Ghitza, He Zhu, Susan Spratt, Marvin Swartz, Paolo Mannelli

**Affiliations:** a Department of Psychiatry and Behavioral Sciences, Duke University Medical Center, Durham, NC, USA; b Department of Medicine, Division of General Internal Medicine, Duke University Medical Center, Durham, NC, USA; c Duke Clinical Research Institute, Duke University Medical Center, Durham, NC, USA; d Center for Child and Family Policy, Sanford School of Public Policy, Duke University, Durham, NC, USA; e National Institute on Drug Abuse, Bethesda, MD, USA; f Department of Medicine, Division of Endocrinology, Duke University Medical Center, Durham, NC, USA

**Keywords:** Comorbidity, Diabetes mellitus, Electronic health records, Mood disorder, Sleep disorder, Substance use disorder

## Abstract

**Background:**

The majority of the U.S. healthcare resources are utilized by a small population characterized as high-risk, high-need persons with complex care needs (e.g., adults with multiple chronic conditions). Substance use disorders (SUDs) and mental health disorders (MHDs) are a driver of poor health and additional healthcare costs, but they are understudied among high-need patients.

**Objective:**

We examine the prevalence and correlates of SUDs and MHDs among adults with high-risk diabetes, who are patients at the top 10% risk score for developing poor outcomes (hospital admission or death).

**Methods:**

A risk algorithm developed from Duke University Health System electronic health records (EHRs) data was used to identify patients with high-risk diabetes for targeting home-based primary care. The EHR data of the 263 patients with high-risk diabetes were analyzed to understand patterns of SUDs and MHDs to inform care-coordinating efforts.

**Results:**

Both SUDs (any SUD 48.3%, alcohol 12.5%, tobacco 38.8%, drug 23.2%) and MHDs (any MHD 74.9%, mood 53.2%, sleep 37.3%, anxiety 32.7%, schizophrenia/psychotics/delusional 14.8%, dementia/delirium/amnestic/cognitive 14.4%, adjustment 9.1%) were prevalent. Overall, 81.7% of the sample had SUD or MHD. Elevated odds of SUD were noted among men (tobacco, alcohol) and those who were never-married (alcohol, cannabis). African-American race (vs. other race/ethnicity) was associated with lower odds of anxiety disorders.

**Conclusion:**

While data are limited to one large academic health system, they provide clinical evidence revealing that 82% of patients with high-risk diabetes had SUD and/or MHD recorded in their EHRs, highlighting a need for developing service models to optimize high-risk care.

## 1. Introduction

In the United States, the top 10% high-need population ranked by their health care expenses paid out of pocket (e.g., adults with multiple chronic conditions) is estimated to account for 61% of total out-of-pocket expenditures, while the lower 50% healthier population accounts for only 2% of the total out-of-pocket expenditures (Cohen, 2014). This high-need population should be researched to inform high-risk care efforts. The U.S. health system has not adequately served adults with the greatest needs (i.e., those with multi-comorbidities) that often receive poorly coordinated care, utilize potentially avoidable hospital care, or have poor outcomes, thereby contributing to spiraling healthcare costs (Hayes et al., 2016a). Behavioral health disorders (BHDs), including substance use disorders (SUDs) and other mental health disorders (MHDs), are an important driver of poorer health and escalating healthcare costs per capita (Hayes et al., 2016b; Laderman, 2015). The lack of BHD screening and implementation of integrated care may contribute to continued high rates of BHD and associated negative outcomes. Persons with comorbid BHD and medical disorders incur much higher healthcare costs (e.g., hospitalization, prescription drugs, clinical visits) than those without such comorbidities (Freeman et al., 2014). Unfortunately, BHDs, especially SUDs, among adults with complex healthcare needs are insufficiently studied to be able to inform targeted screening and care-coordinating efforts (Walter et al., 2017). It is imperative to develop empirical knowledge and better understand patterns of BHDs and risk-management needs for high-cost patients to inform tailored strategies for improving their overall health and reducing healthcare costs per capita (IOM, 2007).

Multi-morbidity has a profound impact on the patient, his/her family, and the U.S. health system. Available data estimated that about 71% of healthcare spending was for patients with multiple chronic conditions, and the number of comorbidities was positively related to poorer health outcomes and higher healthcare costs per capita (Gerteis et al., 2014). Diabetes, a leading cause of death in the United States (Heron, 2016), is among the most prevalent and costly chronic conditions (ADA, 2017a; Ashman et al., 2014; Vijan, 2015). An estimated 30.2 million Americans aged ≥18 years, or 12.2% of the adult population, had diabetes (90–95% of cases with type 2 diabetes) in 2015 (Centers for Disease Control and Prevention (CDC), 2017). The growing aging population, longer life expectancy, and a rising rate of obesity may continue to intensify the epidemic of diabetes, its related chronic conditions (e.g., kidney disease), and societal costs (Gregg and Shaw, 2017; Thorpe et al., 2015). By 2050, one in three U.S. adults is estimated to have diabetes (Boyle et al., 2010). Moreover, national data indicate that multi-comorbidity is common among adults with diabetes: 73% of adults aged 25–44 with diabetes had ≥2 comorbid chronic conditions, which increased to 88% among diabetes adults aged 45–64 (Ashman et al., 2014). Similarly, BHDs are prevalent in the U.S. population. National data estimated that 18% of U.S. adults had a survey-defined MHD in the past year (Center for Behavioral Health Statistics and Quality (CBHSQ), 2016a). An estimated 19% of U.S. adults with a survey-defined MHD had alcohol or drug use disorder and 6% of adults without the survey-defined MHD had alcohol or drug use disorder in the past year (CBHSQ, 2016a).

Individuals with diagnosed diabetes have medical expenditures estimated to be 2.3 times higher than persons without diabetes (ADA, 2015). Economic costs of diabetes in the United States have continued to escalate. For example, between 2007 and 2012, there was a 41% increase in estimated diabetes costs, rising from $174 billion to $245 billion (ADA, 2015). The BHD and diabetes comorbidity represents a ‘double hazard’ in terms of costs, morbidity, and mortality. Both diabetes with complications and BHD (e.g., SUD, mood disorder, schizophrenia) not only are associated with more emergency department (ED) visits and hospitalization readmissions, but also are among the top leading diagnoses among adults who are super-utilizers of hospital care (ADA, 2015; Jiang and Wier, 2010; Jiang et al., 2015). Self-care and good eating habits are crucial for optimal diabetes care management. Having a comorbid SUD may impair self-care and compromise adherence to treatment for diabetes and other comorbidities, thus increasing medical complications or hospital care (Ducat et al., 2014; Ghitza et al., 2013).

Despite substantial health risks from multi-comorbidities, comorbid BHD and diabetes, especially SUDs, are under-recognized and under-treated (Ducat et al., 2014; The Lancet Diabetes Endocrinology, 2015; Walter et al., 2017). In particular, there is a lack of research on SUDs and MHDs for the most-costly, high-risk adults with diabetes. Such high-need, medically comorbid or unstable populations are typically excluded from national surveys of BHDs in the general, non-institutionalized population and clinical trials due to safety consideration or study-specific exclusion criteria (Dennis et al., 2015; Lind, 2011). Therefore, medical record data are needed to characterize such high-cost, vulnerable patients. Given a disproportionally high concentration of healthcare expenditures in a small, but high-need population, we leveraged medical record data to examine the prevalence and correlates of SUDs and MHDs among adults with high-risk diabetes to inform much-needed screening and care-coordination efforts for them.

## 2. Methods

### 2.1. Study sample

Duke University received funds from the Centers for Medicare and Medicaid Services (2012–2016) and the Bristol-Myers Squibb Foundation to augment existing standard of care for adults with type 2 diabetes in community-based medical settings to improve diabetes management (Spratt et al., 2015). Guided by a framework of the spectrum of health and strategies for improvement (Fielding and Teutsch, 2011), an electronic health record (EHR)-based medical risk algorithm was developed using the Duke University Health System EHR data to predict risk for a serious outcome (hospital/ED admission or death) in the subsequent year among adults with type 2 diabetes, which was used to guide the risk-stratified intervention and allocation of available resources (Spratt et al., 2015). The Duke Medicine Enterprise Data Warehouse (EDW) stores the EHR data generated in the healthcare delivery of available patients in the Duke University Health System, including three hospitals and over 200 affiliated primary care and specialty clinics (Horvath et al., 2014). The EDW employs a formal extract, transform, and load procedure to integrate data from source systems on a nightly basis to ensure consistency and quality and to minimize redundancy (Danford et al., 2013).

The risk algorithm was developed initially to predict serious outcomes in 2011 based on the EHR data in 2010, and it was validated by EHR data from 2012 (Spratt et al., 2015). By applying this algorithm to patients’ EHR data, the risk scores for a serious outcome (hospital/ED admission or death) in the coming year were used to identify adults with type 2 diabetes for targeting risk-stratified interventions. Taking into account available resources, adults with a risk score at the top 10% from the risk algorithm were considered high-risk adults for targeting the home-based primary care delivered by a multidisciplinary team (nurse practitioner, social worker, dietitian, and community health worker) over a period of up to 2 years. The home-based care was designed to improve diabetes care, especially for low-income patients with multi-comorbidities (De Jonge et al., 2014; Edwards et al., 2014; Yaggy et al., 2006).

Given that the high-risk population utilizes the majority of health-care resources (Cohen, 2014), this study examined patterns of SUDs and MHDs among high-risk adults with type 2 diabetes that were at the top 10% of the risk score from the risk algorithm. The sample included 263 high-risk adults with type 2 diabetes living in Durham County, NC. The use of their EHR data between January 1, 2012, and June 30, 2016, for this analysis was allowed under a waiver of consent, HIPAA authorization, and the approval from the Duke University Health System Institutional Review Board (IRB). The analysis of those in the lower risk categories was not possible because they were not included in the original IRB approval.

### 2.2. Study variables

Demographic variables, including age, sex, patient-identified race (white/Caucasian, African-American, Asian-American, multiracial), patient-identified ethnicity (Hispanic, non-Hispanic), employment, and marital status, were included as correlates of BHDs.

Diagnostic variables were based on ICD-9-CM and ICD-10-CM codes from the list of discharge or final diagnosis codes for inpatient, out-patient, or ED encounters. Common medical conditions that tended to be associated with diabetes—including diabetic retinopathy, non-traumatic lower extremity amputation, chronic obstructive pulmonary disease (COPD), hypertensive disease, ischemic heart disease, and renal disease—were examined to provide medical profiles (ADA, 2015; Heron, 2016). For example, studies have found a robust association between COPD and type 2 diabetes (Gläser et al., 2015). The number of overall encounters (outpatient, inpatient, ED, other visits) during 01/01/2012–06/30/2016 was included as a control variable to mitigate bias related to health care use and severity of medical conditions (Wu et al., 2015). Diagnosis grouping for each variable was defined to be consistent with those from the Agency for Healthcare Research and Quality (AHRQ)’s Clinical Classification Software (AHRQ, 2016) and Military Health System (MHS)’s surveillance case definitions (MHS, 2015).

To facilitate comparisons with other studies on BHDs, we used the crosswalk of DSM-IV codes to ICD-9-CM codes from the American Psychological Association (APA) Practice Organization for the definition of BHDs that are consistent with major DSM-IV and DSM-5 categories (APA, 2013; APA Practice Organization, 2003). BHDs included alcohol use; tobacco use; drug use (cannabis, cocaine, opioids/heroin, and other drugs); mood; anxiety; schizophrenia/psychotic/delusional; adjustment; personality; somatoform; sleep; eating; dementia/delirium/amnestic/cognitive; disruptive behavioral (attention-deficit/hyperactivity disorder, conduct, oppositional defiant disorder, disruptive behavior, impulse-control); and childhood or developmental (conditions usually diagnosed in childhood) diagnoses.

### 2.3. Statistical analysis

To provide the medical context, we examined frequencies of demographics, healthcare use, and common medical conditions. We then examined the prevalence of BHDs. We conducted separate logistic regression analyses of correlates of SUDs and MHDs, adjusting for the patients’ total number of encounters during 01/01/2012–06/30/2016. To explore whether the presence of SUD and MHD was associated with greater levels of healthcare resource utilization, we conducted ordered logistic regression analysis of the patients’ number of ED/inpatient encounters during 01/01/2012–06/30/2016. Analyses were conducted with Stata 13.1 (StataCorp, 2013).

## 3. Results

### 3.1. Demographic and medical characteristics

The mean age of the sample on January 1, 2012, was 54.6 years (95% confidence interval: 53.2–56.1). Of the sample, 54.0% were women, 76.0% were non-Hispanic African-Americans, 41.8% were disabled, and 38.8% were never married (Table 1). Additionally, 93.5% had at least one ED admission, and 83.7% had at least one inpatient hospitalization logged in the EHRs during 01/01/2012–06/30/2016. Medical conditions were common: diabetic retinopathy (35.7%), non-traumatic lower extremity amputation (17.5%), COPD (27.8%), hypertensive disease (97.0%), ischemic heart disease (49.0%), and renal disease (74.1%).

### 3.2. Prevalence of SUD and MHD diagnoses

**Fig. 1:**
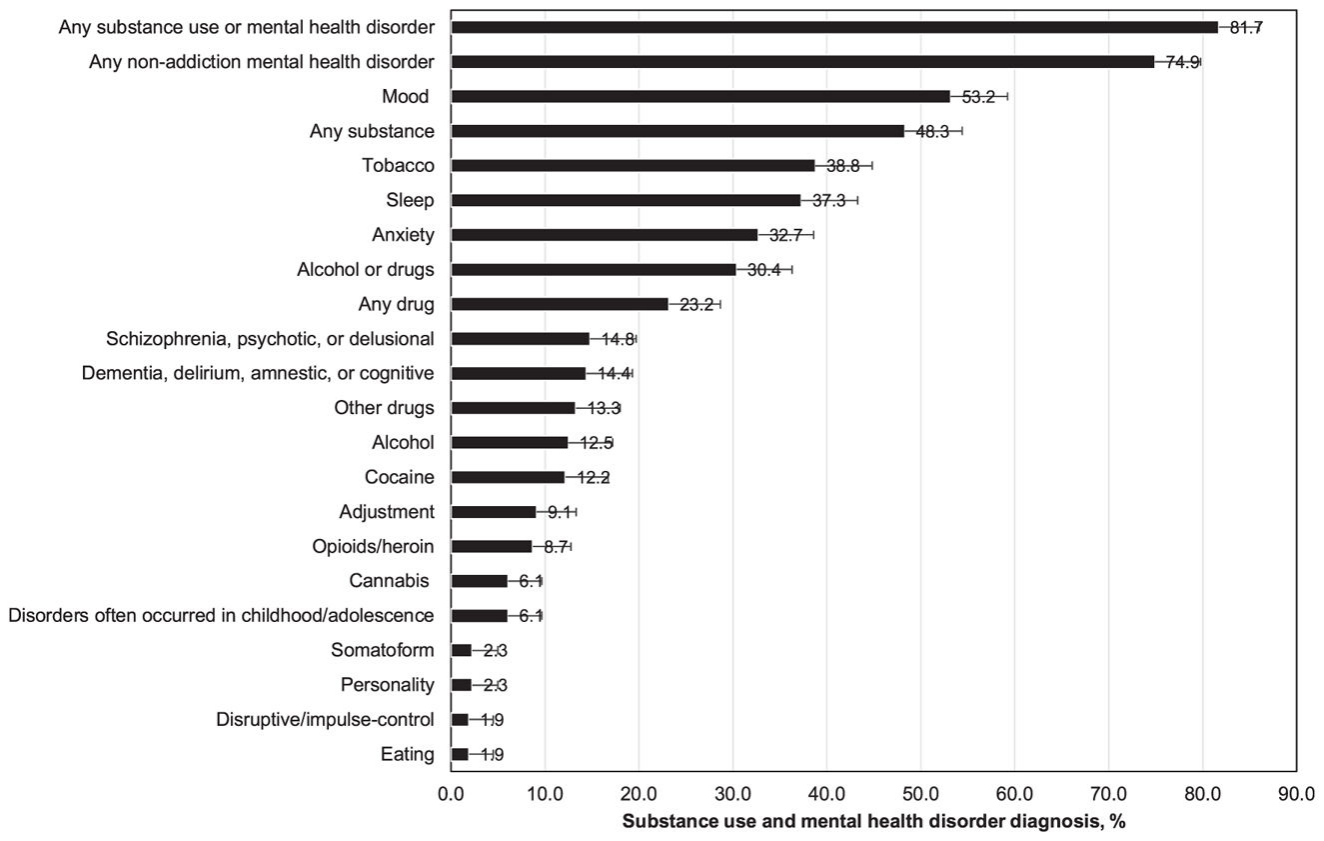
Prevalence of substance use disorder and mental health disorder diagnoses among adults with high-risk diabetes (n = 263).

**Fig. 2:**
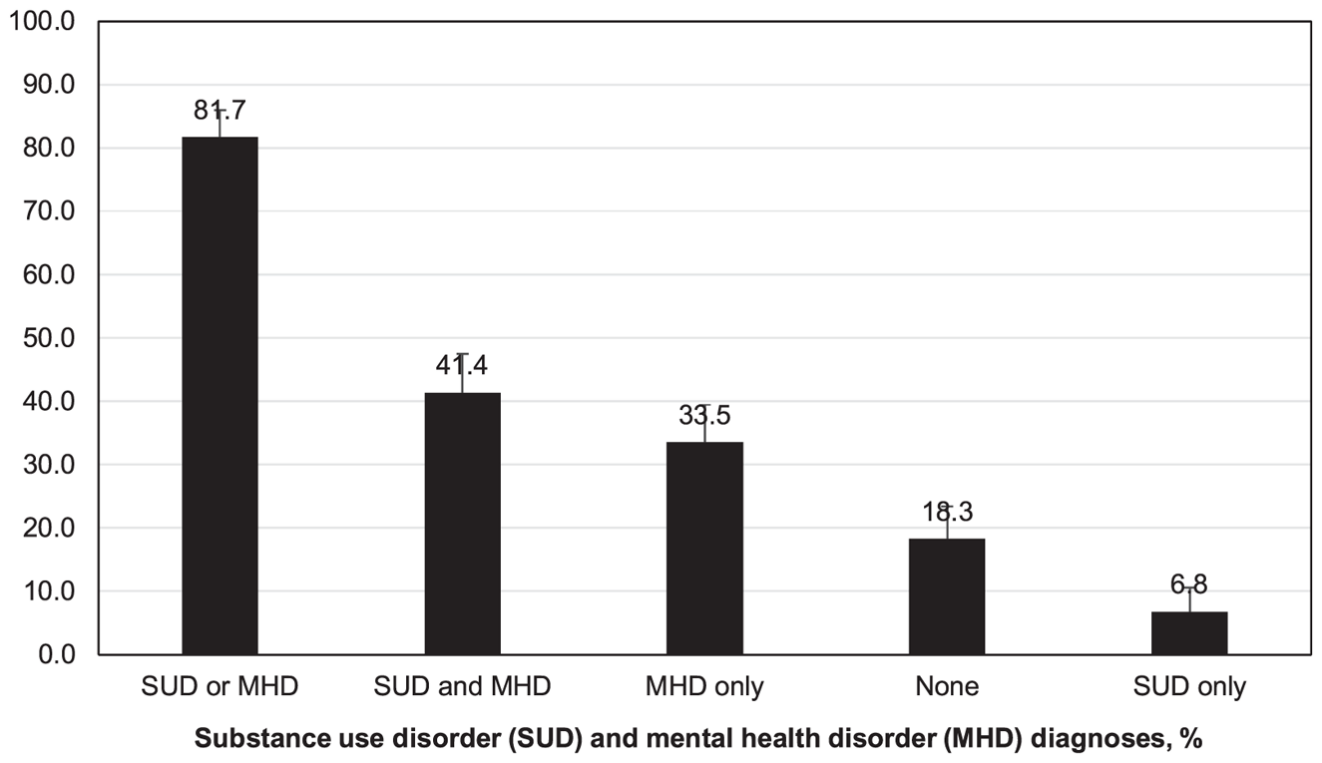
Pattern of comorbid substance use disorder and mental health disorder diagnoses among adults with high-risk diabetes (n = 263).

The prevalence of any SUD in the overall sample was 48.3% (alcohol 12.5%, tobacco 38.8%, and any drug 23.2%) (Fig. 1). Comparatively, common drug use diagnoses were cocaine (12.2%), opioid/heroin (8.7%), and cannabis (6.1%) use disorders. MHD were prevalent (any MHD 74.9%, mood 53.2%, sleep 37.3%, anxiety 32.7%, schizophrenia/psychotics/delusional 14.8%, dementia/delirium/amnestic/cognitive 14.4%, and adjustment 9.1%). Overall, 81.7% of the sample had a BHD: 6.8% had SUD-only, 33.5% had MHD only, and 41.4% had SUD and MHD (Fig. 2). About 86% of diabetes patients with SUD also had MHD, and 55% of diabetes patients with MHD also had SUD. Additional information is reported in the Supplementary Table S1.

### 3.3. Adjusted logistic regression analysis of BHDs

Logistic regression analyses were performed to identify correlates of comparatively common SUD (tobacco, alcohol, cannabis, cocaine, opioid/heroin) and MHD (mood, sleep, anxiety) diagnoses among adults with high-risk diabetes. Each adjusted logistic regression model controlled for the total number of encounters during 01/01/2012–06/30/2016.

#### 3.3.1. SUDs

Compared with male sex, female sex was associated with lower odds of having tobacco and alcohol use disorders. Being married/cohabited (vs. being never-married) was associated with lower odds of having alcohol and cannabis use disorders and being divorced/widowed/separated (vs. being never-married) was associated with lower odds of having cannabis use disorder (Table 2).

#### 3.3.2. MHDs

Compared with being ages 18–49, older ages were associated with lower odds of having mood (for those ages 50–64, ages 65+) and anxiety (for those ages 50–64) disorders. Female sex was associated with elevated odds of having mood and anxiety disorders. Being African-American (vs. other race/ethnicity) was associated with lower odds of having an anxiety disorder. None of the variables examined was associated with a sleep disorder.

### 3.4. BHDs and healthcare utilization

The number of total ED or inpatient encounters by the BHD status is summarized in Table 3. Based on the distribution of the total numbers of ED/inpatient encounters, we used the quartile to create a 4-category variable to denote the level of overall ED/inpatient encounters (from fewer to frequent encounters). Before running each ordered logistic regression model, we first tested the proportional odds assumption or the parallel regression assumption. These tests indicated no violation of the proportional odds assumption for every model examined. The adjusted results showed that each of the SUD and MHD variables were positively associated with elevated levels of ED/inpatient encounters: adjusted ratio ratios ranged from 2.07 for sleep disorder to 6.71 for the presence of 2 or more SUD diagnoses (Table 3).

## 4. Discussion

The United States is facing an epidemic of diabetes: every 21 seconds a person is diagnosed with this disease (ADA, 2017a). Adults with diabetes are at high risk for having serious medical conditions (e.g., kidney or heart disease, lower limb amputation), and muti-morbidity is prevalent (ADA, 2017a; Ashman et al., 2014). SUD and related comorbidities among adults with diabetes can compromise adherence to treatment, exacerbate medical complications, and increase healthcare utilization, but they are under-recognized and under-treated (Ducat et al., 2014; Ghitza et al., 2013; The Lancet Diabetes Endocrinology, 2015). When BHD comorbidities are not treated, the economic costs to the affected families and the health systems are enormous. To mitigate the U.S. health system crisis that is related to substantial healthcare waste and differential concentration of health-care spending by small but high-need patients with multi-comorbidities, the Triple-Aim healthcare reforms promote the development and implementation of patient-centered medical home and other coordinated primary care models in order to provide higher quality care and reduce costs per capita (Berwick et al., 2008; IOM, 2007). Unfortunately, barriers to BHD treatment, especially SUD care, from separately funded and accessed providers can impede integrated care efforts. Hence, identifying feasible strategies to promote the integration of the BHD care into general medical settings and achieve the triple aims for patients affected by multi-comorbidities is becoming a cornerstone of healthcare reform (Laderman, 2015; Powers et al., 2015). This study is among the first to study patterns of SUDs and MHDs among the most-costly, high-risk adults with diabetes that were at the top 10% risk for hospital/ED admission or death. The results from this high-need, but understudied patients help to fill a critical gap in knowledge about their SUD related intervention needs.

### 4.1. Key findings

The key findings concern a high prevalence of BHDs. As many as 82% of these high-risk diabetes patients had one or more BHD present in their EHRs during the study period. The comparison of our findings with other studies is complicated by the fact that U.S. national surveys of BHDs typically use participants’ self-reported information and do not include measures of diabetes comparable to diagnosed diabetes in clinical settings (CBHSQ, 2016a). Data from surveys of samples from the general, non-institutionalized populations are limited to general substance use information. For example, a review showed that among adults with diabetes, approximately 20% were current cigarette smoking and 50%–60% were current alcohol users (Ghitza et al., 2013). A recent review confirmed the scarcity of research on the prevalence of SUDs among adults with diabetes, and the review identified two studies of clinical patients (Walter et al., 2017). Both studies found a similar prevalence of alcohol use disorder (3–4%) and drug use disorder (3–4%) among adult patients with diabetes (Leung et al., 2011; Wu et al., 2015). Based on national survey data, about 8% of U.S. adults had alcohol or drug use disorder in the past year and 12% had nicotine dependence in the past month (CBHSQ, 2016b). In addition, cannabis use disorder (1.4%) was more prevalent than cocaine (0.4%) or prescription opioid (0.8%) use disorder in the general adult population (CBHSQ, 2016b). We found that cannabis use disorder (6.1%) was less prevalent than cocaine (12.2%) or opioid/heroin (8.7%) use disorder. The high prevalence of tobacco use disorder (38.8%) in this clinical sample also has important clinical implications, given the potential synergistic effects of tobacco and diabetes on cardiovascular diseases and mortality (Pan et al., 2015). Thus, the high prevalence of SUD (48.3%) in this sample provides compelling evidence that highlights a need for research on SUDs to better inform SUD treatment strategies for this high-need but overlooked patient group (Engler et al., 2013; Walter et al., 2017).

Another striking finding is the high prevalence of MHDs (any: 74.9%) in this clinical sample. This key finding suggests the existence of substantial challenges to engage these patients in optimal diabetes care and treatment compliance (Ducat et al., 2014). For example, cognition-related conditions, such as schizophrenia/psychotics/delusional (14.8%) and dementia/delirium/amnestic/cognitive (14.4%) diagnoses, may affect patients’ self-care capabilities and adherence to ongoing treatment regimens required for chronic conditions. Additionally, mood (53.2%) and anxiety (32.7%) disorders were more prevalent than other MHDs, and this pattern is consistent with findings from studies of primary care patients with or without diabetes (Katon and Schulberg, 1992; Ornstein et al., 2013; Wu et al., 2015). A meta-analysis found that 17.6% of patients with type 2 diabetes had depression; however, the measures of depression varied considerably across studies (Ali et al., 2006). Overall, MHDs in this high-risk sample were more prevalent than those from a study of 16,243 adults with type 2 diabetes identified from the EHR data (e.g., schizophrenia/psychotics/delusional 3.38%, mood 21.22%, anxiety 13.98%) as well as those from 154,610 adults without type 2 diabetes identified from the EHR data (e.g., schizophrenia/psychotics/delusional 0.98%, mood 9.55%, anxiety 7.53%) (Wu et al., 2015). The high prevalence of BHDs is generally in line with a high-need, high-cost profile, as the prior study included patients with type 2 diabetes in general, regardless of their risk profiles (Wu et al., 2015).

Moreover, we also found a high prevalence of sleep disorders (37.3%) that may have important implications for the management of diabetes and other comorbidities. The presence of sleep disorder may be the manifestation of a multi-morbidity syndrome in this high-risk sample; however, not only it may interfere with treatment compliance, but is also a risk factor for the exacerbation of existing chronic conditions, the occurrence of serious medical complications (e.g., stroke or heart disease), and premature mortality (Ancoli-Israel and Ayalon, 2006; Chawla and Salzman, 2017; Tan et al., 2017). Therefore, improvement of sleep patterns in patients with diabetes may aid the management of other comorbid conditions. Incorporating screening for sleep difficulties and interventions to improve patients’ sleep quality (behavioral therapy, lifestyle intervention, or educational materials) should be part of the treatment plan for high-risk patients with co-morbidities (Chawla and Salzman, 2017; Tan et al., 2017).

### 4.2. Limitations

The findings of this study should be considered within its limitations. The data were from one large academic health system in Durham County, North Carolina, which constrains the generalizability to other settings and regions. It has been well-established that healthcare expenses are highly concentrated in a small population with comorbidities (Berk and Monheit, 1992). For example, 5% of the U.S population in 2002 accounted for 49% of total healthcare expenses (Stanton and Rutherford, 2006). Because existing surveys of SUDs/MHDs are insufficient for studying top-spending individuals with multi-morbidities, it is necessary to use the EHR data to identify such high-need, high-cost healthcare users. The EHR data are collected as part of the routine care that can be influenced by biases (e.g., misclassification, severity of medical conditions, provider specialty), and the recency of EHR-based diagnoses cannot be precisely defined (Wu et al., 2013a,b). All results reported here should be considered with caution and interpreted conservatively within the clinical context.

On the other hand, our high-risk diabetes patients were defined empirically by a mathematical risk algorithm using the EHR data from the Duke University Health System, including demographics, diagnoses, lab results, and medication data known to predict poor outcomes (hospital/ED admission or death) (Spratt et al., 2015). This EHR-data driven approach included multiple covariates/risk factors to improve prediction for developing adverse outcomes in a sequent year within multiple logistic regression frameworks. This regression-type approach may have advantages in identifying ‘clinically high-risk patients’ over a non-empirical approach that used only one or more variable to characterize patients. Our results of medical diagnoses and healthcare utilization indicated a pattern of comorbidities and high-use of hospital care that supports the definition of a selected group of adults with high-risk diabetes. When using a similar approach to compare patterns of BHDs in this sample with results from adult patients with diabetes in general, their distinct patterns in BHDs further confirm a much severe profile of BHDs in high-risk individuals (Wu et al., 2015).

## 5. Conclusion

This study is the first to document a comprehensive pattern of SUD and MHD prevalence among adults with high-risk diabetes. As many as 82% of these high-need patients had BHD logged in their EHRs during a 4.5-year span. BHDs along with heart disease, cancer, trauma, and pulmonary disorders are among the most expensive health conditions in the United States (Stanton and Rutherford, 2006). BHDs, especially SUDs, are understudied and under-treated among the costliest patient groups in primary care (Walter et al., 2017). With the support from Triple-Aim reforms and policy shifts toward value-based care, the time is right for allocating resources to investigate the magnitude of SUDs and associated comorbidities, and implementing Screening, Brief Intervention, and Referral to Treatment (SBIRT) approaches, as well as office-based treatment of alcohol and opioid use disorders for high-need, high-risk patients with diabetes (Levey et al., 2012; Melek et al., 2014; Walter et al., 2017; Wu et al., 2016).

## Supplementary Material

supplement

## Figures and Tables

**Table 1:** Demographic and healthcare characteristics of high-risk diabetes patients: EHR data.^a^

High-risk diabetes patients (Risk score within the top 10%)	Overall (n = 263)	
**Mean**	**Mean**	**95%CI**
Age on 01/01/2012	54.6	53.2–56.1
Number of encounters during the study period^b^	101.3	92.1–110.5
**Proportion**	**%**	**95%CI**
Age group on 01/01/2012		
18–49	33.5	28.0–39.4
50–64	49.0	43.0–55.1
≥65	17.5	13.3–22.6
Sex		
Male	46.0	40.0–52.1
Female	54.0	47.9–60.0
Race/Ethnicity		
Non-Hispanic African American	76.0	70.5–80.8
Other/unknown	24.0	19.2–29.5
Employment		
Disabled	41.8	36.0–47.9
Retired	23.6	18.8–29.1
Other/unknown^c^	34.6	29.1–40.6
Marital status		
Never Married	38.8	33.0–44.8
Married/cohabited	24.0	19.2–29.5
Divorced/widowed/separated	36.9	31.2–42.9
Unknown	0.4	0.1–2.7
Healthcare admission, yes		
ED admission, any	93.5	89.8–96.0
Inpatient hospitalization, any	83.7	78.6–87.7
Medical diagnosis, yes		
Diabetic retinopathy	35.7	30.1–41.8
Non-traumatic lower extremity amputation	17.5	13.3–22.6
Chronic obstructive pulmonary disease	27.8	22.6–33.5
Hypertensive disease^d^	97.0	94.0–98.5
Ischemic heart disease	49.0	43.0–55.1
Renal disease^e^	74.1	68.5–79.1

a Based on electronic health record (EHR) data collected between 01/01/2012 and 06/30/2016.

b The number of encounters was the average number of all encounters per patient in each group, and encounter included ambulatory visits (outpatient visits, telemedicine, etc.), emergency department (ED), inpatient hospitalization, and other visits (e.g., procedure visit) between 01/01/2012 and 06/30/2016.

c Includes those with full- or part-time jobs, the self-employed, the unemployed and those with an unknown employment status.

d Hypertensive disease: essential and secondary hypertension, hypertensive heart disease, hypertensive chronic kidney disease, hypertensive heart and chronic kidney disease.

e Renal disease: nephritis, nephrotic syndrome, or nephrosis.

CI: Confidence interval.

**Table 2:** Adjusted logistic regression of substance use and mental health diagnoses among high-risk diabetes patients: EHR data^a^ (N = 262).

High-risk diabetes patients (Risk score within the top 10%)	Tobacco use disorder		Alcohol use disorder		Cannabis use disorder		Cocaine use disorder		Opioid/heroin use disorder		Mood disorder		Anxiety disorder		Sleep disorder	
							
Adjusted odds ratio (AOR)^b^	AOR	95%CI	AOR	95%CI	AOR	95%CI	AOR	95%CI	AOR	95%CI	AOR	95%CI	AOR	95%CI	AOR	95%CI
Age group on 01/01/2012																
18–49	1.00		1.00		1.00		1.00		1.00		1.00		1.00		1.00	
50–64	0.90	0.50–1.62	2.25	0.89–5.68	0.49	0.16–1.45	0.54	0.24–1.25	1.06	0.38–2.95	**0.33**	**0.17–0.66**	**0.46**	**0.23–0.94**	1.02	0.52–1.99
≥65	0.45	0.16–1.32	0.36	0.03–3.83	––––c	––––––	0.21	0.02–2.56	0.55	0.07–4.22	**0.23**	**0.07–0.70**	0.42	0.12–1.48	1.87	0.60–5.83
Sex																
Male	1.00		1.00		1.00		1.00		1.00		1.00		1.00		1.00	
Female	**0.58**	**0.34–0.98**	**0.14**	**0.05–0.38**	1.17	0.39–3.45	0.62	0.28–1.37	0.75	0.30–1.85	**2.53**	**1.44–4.45**	**5.54**	**2.85–10.79**	1.53	0.86–2.71
Race/Ethnicity																
Non-Hispanic African American	1.00		1.00		1.00		1.00		1.00		1.00		1.00		1.00	
Other/unknown	0.75	0.40–1.39	0.67	0.25–1.80	0.43	0.09–2.04	0.50	0.18–1.41	2.04	0.78–5.37	1.66	0.85–3.25	**2.25**	**1.10–4.60**	1.09	0.55–2.14
Employment																
Disabled	1.00		1.00		1.00		1.00		1.00		1.00		1.00		1.00	
Retired	0.66	0.27–1.59	0.77	0.17–3.50	0.66	0.20–2.10	0.35	0.06–2.15	0.92	0.21–4.02	0.98	0.39–2.43	0.55	0.20–1.54	0.89	0.35–2.26
Other/unknown	0.93	0.49–1.74	1.76	0.68–4.58	––––d	–––––	1.00	0.41–2.44	0.77	0.25–2.32	0.80	0.40–1.60	0.64	0.30–1.34	1.23	0.61–2.48
Marital status																
Never Married	1.00		1.00		1.00		1.00		1.00		1.00		1.00		1.00	
Married/cohabited	1.06	0.55–2.06	**0.27**	**0.09–0.84**	**0.11**	**0.01–0.92**	0.60	0.21–1.70	0.99	0.32–3.06	0.72	0.34–1.50	1.42	0.64–3.15	1.45	0.70–3.01
Divorced/widowed/separated	1.02	0.54–1.92	0.40	0.14–1.12	**0.18**	**0.04–0.92**	1.10	0.43–2.81	1.04	0.34–3.12	0.97	0.49–1.92	1.03	0.49–2.18	1.27	0.63–2.54

a Based on electronic health record (EHR) data collected between 01/01/2012 and 06/30/2016. One patient with unknown marital status was excluded from the analysis.

b Each logistic regression model-controlled variables listed in the first column and log base 10 of the total number of overall encounters (ambulatory, emergency department, inpatient, and other encounters).

c Age groups 50–64 and ≥65 years were combined into one category due to a small sample size.

d Retired and other/unknown employment statuses were combined into one category due to a small sample size.

AOR: Adjusted odds ratio. CI: Confidence interval. Bold face: p < 0.05.

**Table 3:** Adjusted ordered logistic regressions of the number of total emergency department or inpatient encounters in relation to substance use and mental health diagnoses among high-risk diabetes patients: EHR data.

High-risk diabetes patients (Risk score within the top 10%) Behavioral health diagnosis	The number of total emergency department or inpatient encounters Mean (95% CI)	Ordered logistic regression model^a^ (N = 262) AOR (95% CI)
Substance use disorder (tobacco, alcohol, drug), any		
Yes	16.27 (13.59–18.95)	**3.65 (2.25–5.91)**
No	9.34 (7.51–11.16)	Reference
Number of substance use disorders^b^		
2 or more substance use disorder	18.89 (14.94–22.84)	**6.71 (3.57–12.62)**
1 substance use disorder	13.77 (10.21–17.32)	**2.36 (1.35–4.10)**
None	9.34 (7.51–11.16)	Reference
Tobacco use disorder		
Yes	15.76 (12.93–18.60)	**2.76 (1.70–4.47)**
No	10.73 (8.77–12.69)	Reference
Alcohol use disorder		
Yes	16.06 (12.87–19.25)	**4.62 (2.15–9.92)**
No	12.20 (10.37–14.03)	Reference
Cannabis use disorder^c^		
Yes	19.88 (14.72–25.03)	**4.00 (1.48–10.81)**
No	12.22 (10.51–13.93)	Reference
Cocaine use disorder		
Yes	20.53 (16.60–24.47)	**6.16 (2.94–12.89)**
No	11.60 (9.84–13.36)	Reference
Opioid/heroin use disorder		
Yes	20.09 (11.19–28.99)	**2.70 (1.21–6.02)**
No	11.98 (10.40–13.55)	Reference
Mood disorder		
Yes	14.89 (12.82–16.96)	**2.67 (1.63–4.35)**
No	10.17 (7.60–12.74)	Reference
Anxiety disorder		
Yes	18.05 (15.09–21.00)	**4.50 (2.61–7.76)**
No	10.08 (8.20–11.96)	Reference
Sleep disorder		
Yes	16.39 (13.12–19.65)	**2.07 (1.28–3.33)**
No	10.48 (8.78–12.19)	Reference

Based on electronic health record (EHR) data collected between 01/01/2012 and 06/30/2016. One patient with unknown marital status was excluded from the analysis. AOR: Adjusted odds ratio. CI: Confidence interval. Bold face: p < 0.05.

a Each ordered logistic regression model of ED/inpatient encounters in relation to a behavioral health diagnosis controlled for age on 01/01/2012, sex, race/ethnicity, employment, and marital status variables. Dependent variable was a 4-category variable of the number of total ED or inpatient encounters, representing four quartiles of the distribution (0–3, 4–9, 10–18, 19–108 encounters).

b The number of substance use disorders counted tobacco, alcohol, cannabis, cocaine, opioid/heroin, and other drug use disorders.

c Age groups 50–64 and ≥65 years were combined into one category and retired and other/unknown employment status were combined into one category due to a small sample size.

## Appendix A. Supplementary data

Supplementary data associated with this article can be found, in the online version, at https://doi.org/10.1016/j.drugalcdep.2018.01.008.
