# Supplementary material for: Substance use disorders and medical comorbidities among high-need, high-risk patients with diabetes
Source: Drug Alcohol Depend. Author manuscript; Available in PMC 2018 May 18. (PMC5959045; doi:10.1016/j.drugalcdep.2018.01.008)
Supplement: supplement [file NIHMS965661-supplement.docx]

**Supplementary Material for the Article:**

**Substance use disorders and medical comorbidities among high-need,**

**high-risk patients with diabetes**

**This material supplements, but does not replace, the peer-reviewed paper in**

***Drug and Alcohol Dependence*.**

Li-Tzy Wu^1,2,3,4^, Udi E. Ghitza^5^, He Zhu^1^, Susan Spratt^6^, Marvin Swartz^1^, Paolo Mannelli^1^

**^1^** Department of Psychiatry and Behavioral Sciences, Duke University Medical Center, Durham, NC, USA

**^2^** Department of Medicine, Division of General Internal Medicine, Duke University Medical Center, Durham, NC, USA

**^3^** Duke Clinical Research Institute, Duke University Medical Center, Durham, NC, USA

**^4^** Center for Child and Family Policy, Sanford School of Public Policy, Duke University, Durham, NC, USA

**^5^** National Institute on Drug Abuse, Bethesda, MD, USA

**^6^** Department of Medicine, Division of Endocrinology, Duke University Medical Center, Durham, NC, USA

**Correspondence:**

Li-Tzy Wu

Department of Psychiatry and Behavioral Sciences, School of Medicine, Duke University Medical Center, Box 3903, Durham, NC 27710, USA

Phone: 919-668-6067

litzy.wu@duke.edu

##### Table S1. Substance use and mental health disorders diagnoses among high-risk diabetes patients: EHR data¹

| **High-risk diabetes patients**  **(Risk score within the top 10%)** | **Overall**  **(n=263)** | |
| --- | --- | --- |
| **Substance use and mental health disorders diagnosis, yes** | **%** | **95%CI** |
| Alcohol | 12.5 | 9.0-17.2 |
| Tobacco | 38.8 | 33.0-44.8 |
| Drugs, any² | 23.2 | 18.5-28.7 |
| Cannabis | 6.1 | 3.7-9.7 |
| Cocaine | 12.2 | 8.7-16.7 |
| Opioids/heroin | 8.7 | 5.9-12.8 |
| Other drugs | 13.3 | 9.7-18.0 |
| Alcohol or drugs | 30.4 | 25.1-36.3 |
| Any substance (alcohol, tobacco, or drugs) | 48.3 | 42.3-54.4 |
| Mood | 53.2 | 47.1-59.2 |
| Sleep | 37.3 | 31.6-43.3 |
| Anxiety | 32.7 | 27.3-38.6 |
| Schizophrenia, psychotic, or delusional | 14.8 | 11.0-19.7 |
| Dementia, delirium, amnestic, or cognitive | 14.4 | 10.7-19.3 |
| Adjustment | 9.1 | 6.2-13.3 |
| Disorders often occurred in childhood or adolescence | 6.1 | 3.7-9.7 |
| Personality | 2.3 | 1.0-5.0 |
| Somatoform | 2.3 | 1.0-5.0 |
| Eating | 1.9 | 0.8-4.5 |
| Disruptive/impulse-control ^3^ | 1.9 | 0.8-4.5 |
| Any non-addiction mental health disorder diagnosis^4^ | 74.9 | 69.3-79.8 |
| Any substance use or mental health disorder diagnosis^5^ | 81.7 | 76.6-86.0 |

¹ Based on electronic health record (EHR) data collected from between 01/01/2012 and 06/30/2016. ² Drugs included cannabis, opioids/heroin, cocaine, and other drugs. ³ Disruptive/impulse-control diagnoses included attention-deficit/hyperactivity disorder (ADHD), conduct disorder (CD), oppositional defiant disorder (ODD), disruptive behavior disorder (DBD), and impulse-control disorders. ^4^Any non-addiction mental health disorder diagnosis included mood, anxiety, schizophrenia/psychotic, adjustment, personality, somatoform, eating, sleep, Disruptive/impulse-control, dementia/delirium/amnestic/cognitive, and disorders often occurred in childhood or adolescence (e.g., developmental diagnoses). ^5^Any substance use or mental health disorder diagnosis included any substance and non-addiction mental health disorders.

CI: Confidence interval.
